# Role of 11β-Hydroxysteroid Dehydrogenase and Mineralocorticoid Receptor on Alzheimer’s Disease Onset: A Systematic Review

**DOI:** 10.3390/ijms26031357

**Published:** 2025-02-06

**Authors:** Mariangela Di Vincenzo, Pamela Pellegrino, Genny Schiappa, Anna Campanati, Valerio Del Vescovo, Silvia Piccirillo, Patrizia Ambrogini, Giorgio Arnaldi, Monia Orciani

**Affiliations:** 1 Department of Clinical and Molecular Sciences—Histology, Università Politecnica delle Marche, 60126 Ancona, Italy; m.divincenzo@pm.univpm.it (M.D.V.); p.pellegrino@univpm.it (P.P.); gennyschiappa@tim.it (G.S.); valerio9692@gmail.com (V.D.V.); 2 Department of Clinical and Molecular Sciences—Dermatological Clinic, Università Politecnica delle Marche, 60126 Ancona, Italy; a.campanati@univpm.it; 3 Department of Biomedical Sciences and Public Health—Pharmacology, Università Politecnica delle Marche, 60126 Ancona, Italy; s.piccirillo@univpm.it; 4 Department of Biomolecular Sciences, University of Urbino Carlo Bo, 61029 Urbino, Italy; 5 Department of Health Promotion, Mother and Child Care, Internal Medicine and Medical Specialties (ProMISE) “G. D’Alessandro”, University of Palermo, 90127 Palermo, Italy; giorgio.arnaldi@unipa.it

**Keywords:** cortisol, Alzheimer’s disease, androgen receptor

## Abstract

The role of 11β-HSD1 in Alzheimer’s disease (AD) has garnered significant attention due to its involvement in glucocorticoid metabolism, neuroinflammation, and cognitive decline. This review explores the current understanding of 11β-HSD1 in AD, examining genetic, preclinical, and clinical research. Genetic studies have identified 11β-HSD1 polymorphisms that may influence AD risk, although findings remain inconsistent. Mechanistically, 11β-HSD1 promotes neurodegeneration through the dysregulation of glucocorticoid activity, contributing to hippocampal atrophy, amyloid plaque formation, and tau pathology. Preclinical studies have shown that 11β-HSD1 inhibitors offer neuroprotective effects, including enhanced cognitive function, reduced inflammation, and improved mitochondrial activity. However, clinical trials, including those involving ABT-384 and Xanamem, have produced mixed results, with no substantial cognitive improvements despite effective enzyme inhibition. These inconsistencies highlight the complexity of AD and the challenges in translating preclinical findings into clinical outcomes. Moreover, while 11β-HSD1 inhibition holds therapeutic potential, other strategies targeting neuroinflammation, autophagy, and glucocorticoid signaling are also being explored. Ongoing research is focusing on optimizing 11β-HSD1 inhibitors, identifying biomarkers for patient selection, and investigating combination therapies to enhance treatment efficacy. Ultimately, 11β-HSD1’s role in AD presents a promising therapeutic target, but further studies are required to fully understand its potential in managing the disease.

## 1. Introduction

Alzheimer’s disease (AD) is a progressive neurodegenerative condition and the leading cause of dementia, primarily affecting older adults [1]. It is marked by a gradual decline in cognitive abilities, memory loss, and behavioral or personality changes. Key features of Alzheimer’s include the accumulation of amyloid-beta plaques and neurofibrillary tangles made of tau protein in the brain. These pathological alterations result in neuronal damage and brain shrinkage, especially in areas involved in memory and learning, such as the hippocampus.

Different hormones and neuropeptides are involved in neurodegenerative diseases like AD as their dysregulation can worsen cognitive decline and neurodegeneration. Among the others, insulin resistance links metabolic issues to cognitive impairment in AD [2]; reduced levels of orexin, a neuropeptide that regulates wakefulness, is associated with cognitive deficits and may accelerate neurodegeneration [3,4]; and somatostatin and CRH influence cognitive function and are altered in AD [5,6], with CRH being linked to amyloid-beta accumulation.

A pivotal role is played by hypothalamic–pituitary–adrenal (HPA) axis in stress responses, influencing regions like the prefrontal cortex (PFC), hippocampus, and amygdala [7,8]. Chronic stress activates the HPA axis, leading to the secretion of glucocorticoids (GCs), which can alter brain function. The dysregulation of the HPA axis, including impaired glucocorticoid receptor (GR) function, is linked to cognitive decline in AD. Overexposure to GCs, often associated with prolonged stress or disorders such as Cushing’s syndrome (CS), in the PFC and hippocampus may worsen cognitive deficits in AD. 

An excess of cortisol also results in exacerbating inflammation, oxidative stress, and amyloid-beta accumulation, thus accelerating the progression of Alzheimer’s disease [9,10].

The connection between AD and cortisol has traditionally been viewed as a direct interaction of the hormone with its receptor, leading to impaired neurogenesis. However, cortisol may also affect AD progression through the mineralocorticoid receptor (MCR), specifically via the 11beta-Hydroxysteroid dehydrogenase (11β-HSD) pathway.

The type I isoform (11β-HSD1) is a bidirectional enzyme but acts predominantly as an oxidoreductase to form the active glucocorticoids cortisol, while the type II enzyme (11β-HSD2) acts unidirectionally, producing inactive 11-keto metabolites [11]. 

**Figure 1:**
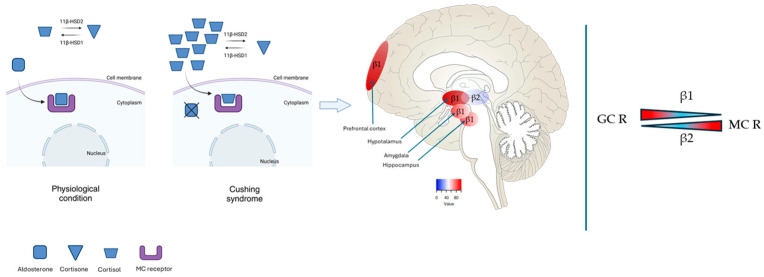
**Left panel.** The image describes how in physiological condition 11β-HSD2 is able to drive the conversion of cortisol in cortisone allowing aldosterone to bind its receptor; the level of cortisol in Cushing’s syndrome is so high that 11β-HSD2 cannot buffer the presence of the active form of the hormone that remains available for the binding with mineralocorticoid (MC) receptor, leading to neural damage. β1=11β-HSD1; β2=11β-HSD2. **Right panel.** Different affinity of 11β-HSD1 and 11β-HSD2 for glucocorticoid (GR) receptor and mineralocorticoid (MC) receptor.

11β-HSD2 is a high-affinity NAD-dependent dehydrogenase that protects the mineralocorticoid receptor from glucocorticoid excess in still physiological condition; in conditions of chronically elevated cortisol levels, such as in CS, 11β-HSD2 is no longer able to buffer the presence of active cortisol and the overactivation of the mineralocorticoid receptors by cortisol has been linked to increased oxidative stress and neuronal damage (Figure 1) [12]. The dysregulation of the mineralocorticoid receptor pathway may play a role in neurodegenerative processes, suggesting a non-traditional connection between excessive cortisol signaling and the progression of AD.

Here, we consider and discuss the published articles focusing on the effects of cortisol excess on AD, specifically via 11β-HSD1 on the approach developed by Arksey and O’Malley [13].

## 2. Methods

This review was based on the approach developed by Arksey and O’Malley [13], including five essential steps: the identification of the research question; the identification of appropriate studies; the selection of studies; the tracking of data; and the collection, summarization, and reporting of results. The Preferred Reporting Items for Systematic Reviews and Meta-Analysis (PRISMA) extension for scoping review criteria was used to guide the conduction and reporting of the review. This review was registered in the International Prospective Register of Systematic Reviews (PROSPERO) with the registration number “CRD42023420245”.

### 2.1. Identification of the Research Question

The research question was formulated during a collaborative brainstorming session with the entire research team, which consisted of three biologists specializing in neurogenesis, three biologists with expertise in glucocorticoid excess, and one endocrinologist with expertise in Cushing’s syndrome. In the initial meeting, the team developed the research question and subsequently outlined the research strategy.

The question posed was: “Does glucocorticoid excess influence neurogenesis in Alzheimer’s patients via the 11β-HSD activity, and if so, how?”

### 2.2. Study Selection Process

A comprehensive systematic review of studies on this topic was conducted using three electronic medical databases—PubMed, Web of Science, and SCOPUS. Search terms were chosen to identify studies that explored the relationship between the 11β-HSD activity and Alzheimer’s disease.

The keywords used were as follows:

- 11β-HSD and Alzheimer disease;
- 11beta-HSD and Alzheimer disease;
- 11beta-hydroxysteroid dehydrogenase and Alzheimer disease.

Selected databases were searched from their respective inceptions. 

In this first phase, a total of 163 records were retrieved from the selected database (Table 1). 

After removing duplicates, 62 records remained. Relevant studies were then identified through a three-phase process. In the first phase, two researchers (MDV, PP) independently screened articles based on their titles, narrowing the selection to 26. Any disagreements were resolved by consulting a senior researcher (MO). In the second phase, abstracts were assessed, with all team members independently reviewing each one. Discrepancies were resolved by unanimous agreement within the research group. As a result, 2 articles were excluded, leaving 24 for full-text analysis.

The third phase involved a thorough evaluation of the full text of the 24 selected papers on the 11β-HSD and Alzheimer’s disease. To be included in the review, studies needed to directly address the research question, and all included studies had to have an available abstract. There were no limitations on study design; in vitro and in vivo pre-clinical trials, controlled clinical trials, case–control studies, cross-sectional studies, and reviews were all considered. By manually checking the references, two more elements were included. Ultimately, twenty-six studies were included for full-text analysis on the 11β-HSD and Alzheimer’s disease, but six items were definitively excluded.

## 3. Results and Discussion

**Figure 2:**
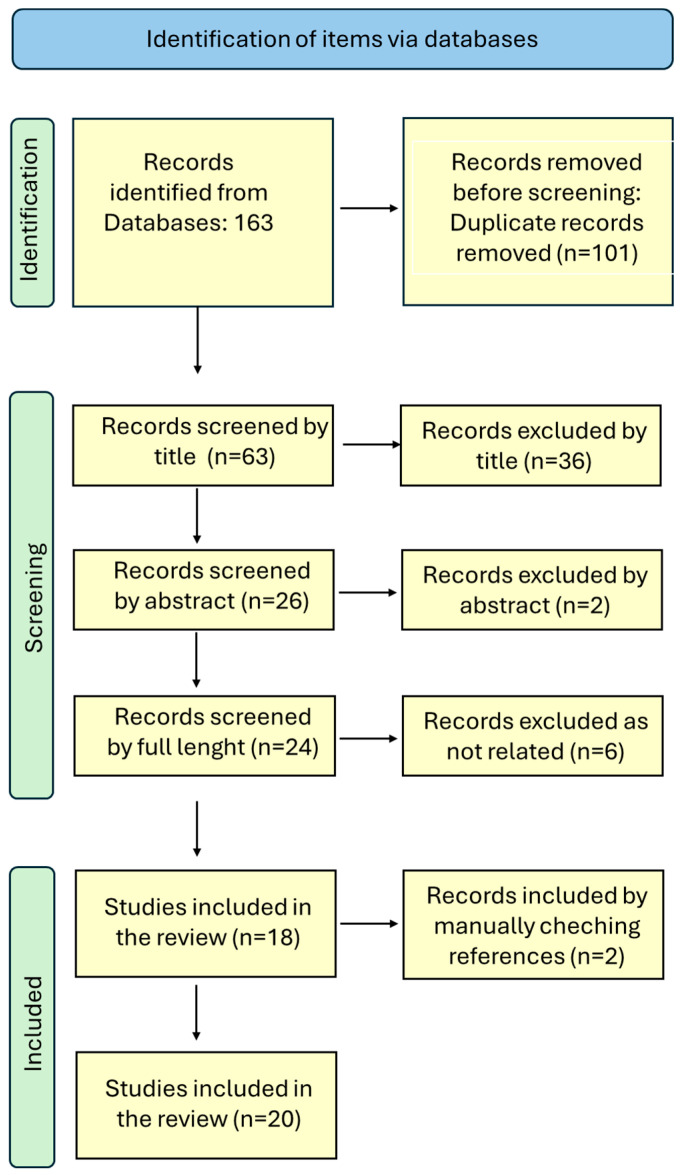
Flow diagram of the items’ selection. Freely inspired and modified by PRISMA 2020 flow diagram. For more information, visit: http://www.prisam-statement.org/ (accessed on 18 October 2024).

The flowchart of the PRISMA study is shown in Figure 2.

Starting from the initial selection of 163 records, our search identified 62 records after removing duplicates. After reviewing titles and abstracts, 38 citations were dropped (reports not strictly related to the item retrieved), and 24 were evaluated for full-text eligibility. Two more items were added by manual checking of the references, for a total of 26 included studies, but after full-text evaluation, 20 items were finally reviewed. 

We divided the items according to the following sections:

- Section 1 focuses on the biology and genetics of 11β-HSD1.
- Section 2 presents the preclinical groundwork that supports therapeutic inhibition.
- Section 3 addresses clinical trials directly, separating successes (Xanamem) from challenges (ABT-384).
- Section 4 highlights innovations in inhibitor design and their effects.

This organization mirrors the drug development pipeline:

- From basic biology → preclinical studies → clinical applications → novel designs and disease-modifying effects.

### 3.1. Role of 11β-HSD1 and HSD11B1 Polymorphisms in AD Pathophysiology

This section explores the biological and genetic foundations of 11β-HSD1 in Alzheimer’s disease, focusing on its regulation, genetic variations, and their implications for disease progression.

#### 3.1.1. Basic Research on 11β-HSD1 Function

- Herbert and Lucassen [14] reported that 11β-HSD1 inhibition protects neurons and improves cognition in aging models, suggesting that excessive glucocorticoid activity is neurotoxic. Conversely, they found that 11β-HSD2 induction plays a protective role in cerebellar neurons. These findings highlight the dual roles of 11β-HSD enzymes in regulating glucocorticoid metabolism and their potential as therapeutic targets.
- Surprisingly, the authors later pointed out a study by de Quervain Dominique [15], which suggested that a rare variant of the HSD11B1 gene associated with reduced 11β-HSD1 activity increases AD risk sixfold. This finding contrasts with the prevailing understanding of 11β-HSD1 as harmful in AD. Upon further review of this study, they clarified that the observed effects were context-dependent: in cell homogenates, 11β-HSD1 favored dehydrogenase activity (converting active cortisol to inactive cortisone), whereas in cell cultures, the enzyme primarily acted as a reductase, regenerating active cortisol. This discrepancy underscores the complexity of 11β-HSD1’s role in AD and the need for further study.

These conflicting findings underscore the need for further research to clarify the enzyme’s precise role in the pathophysiology of AD. It is essential to explore how various genetic, environmental, and biochemical factors influence 11β-HSD1 activity and determine whether the inhibition or activation of this enzyme is more beneficial, based on specific disease stages or patient subgroups. 

#### 3.1.2. Polymorphisms of the HSD11B1 Gene

- rs846911 polymorphism: A rare A allele of this polymorphism was found in 2.9% of AD patients compared to 0.5% of controls. This suggests that certain genetic variants of 11β-HSD1 might predispose individuals to AD [15].
- 83,557 insA polymorphism: In a study by Smit et al., the percentage of this variant was high (34.6% heterozygous and 4.8% homozygous) in the study groups consisting in 6105 subjects, but no significant correlation was found with dementia incidence. This suggests that not all genetic variations of 11β-HSD1 are equally impactful in AD [16,17].
- rs12086634-G/T, rs846910-A/G: Deary et al. tested the hypothesis that these polymorphisms were associated with lifetime cognitive change in humans. They evaluated the presence of polymorphisms in 194 participants at age 11 and age 79. They found no association between *HSD11B1* SNPs and cognitive variation with aging in a Scottish cohort, concluding that further studies in Alzheimer’s disease in different populations were warranted [18].

The link between 11β-HSD1 polymorphisms and Alzheimer’s disease (AD) risk remains unclear, with prior studies offering conflicting results. This variation highlights the complexity of genetic contributions to AD and underscores the need for further investigation to define the role of 11β-HSD1 variants in disease susceptibility.

These inconsistencies emphasize the importance of conducting larger and more robust studies, involving diverse populations, to gain a deeper understanding of how 11β-HSD1 genetic variants influence AD. Given the complex interactions between genetic, environmental, and epigenetic factors, future research should consider these polymorphisms alongside other potential risk factors, with a focus on identifying subgroups more genetically predisposed to the disease. Such an approach could ultimately lead to improved diagnostic tools and more targeted interventions for those at risk of developing Alzheimer’s.

### 3.2. Preclinical Evidence Supporting 11β-HSD1 Inhibition

- This section synthesizes evidence from animal and cellular preclinical studies, underlining the neuroprotective potential of 11β-HSD1 inhibition in AD and suggesting that modulating glucocorticoid metabolism may alleviate AD-related damage. These studies offer valuable insights into the role of 11β-HSD1 in regulating local glucocorticoid levels and its impact on neurodegeneration. Li et al. [19] demonstrated that in animal models of AD, pharmacological inhibition or genetic knockdown of 11β-HSD1 prevented cognitive impairment caused by excessive local steroid action. This study linked excessive glucocorticoid activity to hippocampal damage and validated 11β-HSD1 inhibition as a neuroprotective strategy.
- Chowdhury et al. [20] examined the overexpression of 11β-HSD1 in cell models, which resulted in reduced cell proliferation and increased apoptosis, suggesting that hyperactivity of this enzyme directly contributes to neuronal loss.
- Wang et al. [21] investigated the effects of Akebia saponin D (ASD) in an amyloid-beta rat model of AD. ASD reversed corticosterone increases via HPA axis regulation, though it did not directly inhibit 11β-HSD1. These findings indicate that reducing corticosterone levels may have therapeutic benefits in AD.

Collectively, these studies underscore the potential of 11β-HSD1 inhibition as a neuroprotective approach in AD. However, while preclinical evidence supports its promise, there remain critical questions regarding the long-term efficacy, safety, and specificity of such interventions. Further research is needed to understand how 11β-HSD1 inhibition interacts with other neurobiological pathways in AD, and whether these findings can be translated into effective clinical treatments. The complexity of glucocorticoid signaling in the brain, coupled with the multifactorial nature of AD, calls for a more nuanced approach to targeting 11β-HSD1 and its associated pathways.

### 3.3. Clinical Trials of 11β-HSD1 Inhibitors

Human studies are critical to translating preclinical findings into viable treatments. This section focuses on the outcomes of clinical trials for ABT-384 and Xanamem (UE2343). More details about these molecules are reported in Table 2.

#### 3.3.1. ABT-384

- Phase II Trial: ABT-384, a selective 11β-HSD1 inhibitor, was tested in AD patients to assess its ability to reduce intracellular cortisol and improve cognition. While the compound successfully inhibited 11β-HSD1 in the brain, no significant cognitive improvements were observed, as measured by the Alzheimer’s Disease Assessment Scale—Cognitive (ADAS-Cog). The trial was terminated early for futility, highlighting the challenges of targeting glucocorticoid metabolism in AD [22].
- Katz et al. [23]: This study confirmed that ABT-384 effectively inhibited both peripheral and central 11β-HSD1 at low doses (1–2 mg). However, the lack of clinical efficacy despite strong enzymatic inhibition contrasts with the hypothesis that cortisol is deregulated.

#### 3.3.2. Xanamem (UE2343)

- Webster et al. [24]: This study demonstrated that Xanamem, another selective 11β-HSD1 inhibitor, effectively penetrated the brain, showing favorable safety and pharmacokinetics in early trials.
- Taylor et al. [25]: In a Phase II trial, Xanamem improved cognition in patients with high levels of pTau181, a biomarker of tauopathy. This suggests that Xanamem may be particularly effective in tau-driven subtypes of AD.
- Dodd et al.: In a Phase II, Xanamem did not achieve statistically significant results at a dose of 10 mg/day and new results about 20 mg daily dose to reach cognitive protection have to be published [12].

Despite strong evidence of enzymatic inhibition, the lack of significant cognitive benefits in clinical trials highlights the critical gap in understanding the mechanisms by which 11β-HSD1 inhibitors may produce their effects. While preclinical data suggest that targeting glucocorticoid metabolism could influence neuroinflammation and cognitive function, the absence of clear cognitive improvements in clinical settings implies that the underlying pathways are more complex than initially thought. This discrepancy underscores the urgent need for further mechanistic studies to clarify how 11β-HSD1 inhibition interacts with other brain processes, particularly in AD.

In addition, it should be considered that in AD the progression occurs through distinct stages, each characterized by specific pathophysiological alterations, with early intervention offering better therapeutic potential due to less severe neurodegeneration. In contrast, treatments administered during advanced stages, marked by pronounced cognitive impairment and extensive neurodegeneration, are likely to exhibit limited effectiveness. Dosing strategies should be adjusted according to disease stage, with higher doses in later stages and lower doses in earlier stages. Tailoring treatments according to the stage of AD, alongside optimizing dosing regimens, could improve clinical trial outcomes and enhance the efficacy of therapies like 11β-HSD1 inhibitors.

In conclusion, although the potential of 11β-HSD1 inhibitors remains, the challenge lies in determining why these treatments fail to deliver the expected cognitive improvements in clinical trials. Future research must focus on uncovering the molecular mechanisms at play and identifying validated biomarkers to select specific subgroups of AD patients who might benefit most from these therapies. A deeper understanding of these mechanisms is essential to bridging the gap between preclinical success and clinical efficacy in AD treatment.

### 3.4. Novel and Optimized 11β-HSD1 Inhibitors

Significant efforts have been made to design next-generation inhibitors with improved potency, selectivity, and brain penetration.

- Leiva et al. [26]: The authors of this study developed inhibitors featuring unexplored pyrrolidine-based polycyclic substituents, demonstrating a high affinity for 11β-HSD1. This compound improved memory and learning in animal models of age-related cognitive decline, reduced neuroinflammation, and decreased oxidative stress.
- Puigoriol-Illamola et al. [27,28]: In this study, researchers developed RL-118, a pyrrolidine-based inhibitor, which alleviated cognitive impairments in aging mice by promoting autophagy and improving mitochondrial function. RL-118 boosted Beclin1 and LC3B levels, facilitating the clearance of tau. It also reduced glucocorticoid receptor expression and systemic glucocorticoid levels, demonstrating broad neuroprotective effects. Moreover, the study found that inhibiting 11β-HSD1 with RL-118 mitigated the harmful effects caused by chronic mild stress, including epigenetic and cognitive disruptions. This suggests that reducing glucocorticoid excess could be a promising therapeutic approach for age-related cognitive decline and Alzheimer’s disease.
- Sooy et al. [29]: In this study, the authors evaluated UE2316 (more details on Table 2), which reduced amyloid plaque burden in Tg2576 mice and improved memory. This compound lowered glucocorticoid levels in the brain, restoring the balance between glucocorticoid and mineralocorticoid receptor activity. In addition, in [29], the chronic administration of this inhibitor significantly reduced amyloid plaques in the cortex and amygdala of Tg2576 mice. This was associated with improvements in memory and synaptic integrity. Mohler et al. [30] characterized two novel and selective HSD1 inhibitors, A-918446 and A-801195, and learning, memory consolidation, and recall were evaluated as well as CREB, a transcription factor involved in cognition. Treatments inhibited cortisol production in the ex vivo assay by ∼35–90%, significantly improving short-term memory in rats.
- Canet et al. [7] focused on the role of early dysregulation of the hypothalamic–pituitary–adrenal axis (HPA axis or stress axis) observed in AD patients and the inhibition of 11β-HSD1 was able to partially restore it. The authors summarized all the different 11β-HSD1 inhibitors, demonstrating that all of them, according to the different animal models, lead to memory improvement and Aβ plaques decrease. Other effects of 11β-HSD1 on the HPA axis were reported by Seckl [31]. In mice, the inhibition of 11β-HSD1 disrupted glucocorticoid feedback regulation in the HPA axis, leading to reduced glucocorticoid levels and the compensatory activation of the axis. This resulted in elevated ACTH and adrenal activity. In humans, 11β-HSD1 inhibitors typically do not alter cortisol but cause modest increases in ACTH and adrenal products like DHEA, reflecting the compensatory activation of the HPA axis. Indeed, elevated ACTH and DHEA are useful biomarkers of effective 11β-HSD1 inhibition in humans.

While the studies reviewed demonstrate the promising therapeutic potential of 11β-HSD1 inhibitors in addressing key AD pathologies, including amyloid plaques, tau tangles, and neuroinflammation, several challenges remain. Despite encouraging preclinical results, the translation of these findings into effective human therapies requires careful optimization. Key considerations include enhancing the brain penetration of these inhibitors, evaluating their long-term efficacy, and ensuring their safety in clinical settings. Furthermore, the modulation of the hypothalamic–pituitary–adrenal (HPA) axis and glucocorticoid levels represents a complex approach that may influence multiple aspects of AD pathophysiology. However, the intricate interactions between glucocorticoid signaling, cognitive function, and neurodegeneration must be thoroughly understood before these inhibitors can be considered viable clinical treatments. 

## 4. Limitations of the Study and the Selection of Articles

A limitation of this study lies in the selection process, which could introduce bias. The focus on studies directly addressing 11β-HSD1 and Alzheimer’s disease may have excluded relevant research on indirect pathways or complex interactions. Additionally, limiting the search to three databases could have missed valuable studies from other sources. Subjective interpretation during study evaluation and reliance on published studies may also lead to the exclusion of valuable insights and publication bias, potentially skewing the findings.

## 5. Conclusions

Research on 11β-HSD1 in Alzheimer’s disease (AD) highlights its role in glucocorticoid regulation, neuroinflammation, and cognitive decline, as summarized in Table 3. Genetic studies suggest links to AD risk, though the results are mixed. Preclinical data support the potential of 11β-HSD1 inhibitors, like RL-118 and UE2316, in reducing neuroinflammation and improving memory. However, clinical trials with ABT-384 and Xanamem show limited cognitive benefits, emphasizing the complexity of AD that involves multiple interconnected pathways. While 11β-HSD1 inhibition may address one aspect of the disease—namely, modulating glucocorticoid metabolism—it is unlikely to be sufficient on its own to tackle the broader range of pathophysiological processes involved in AD. A more effective approach could involve targeting multiple pathways simultaneously, potentially in combination with other treatments that address neuroinflammation, autophagy, and neuronal health. This multi-target strategy may offer a more comprehensive therapeutic approach and improve outcomes in AD.

## Figures and Tables

**Table 1:** Number of records found in the electronic medical databases, namely PubMed, Web of Science, and SCOPUS, according to different keywords.

	N. Records		
	PubMed	Web of Science	Scopus
**11β-HSD and Alzheimer disease**	22	8	7
**11beta-HSD and Alzheimer disease**	22	9	7
**11beta-hydroxysteroid dehydrogenase and Alzheimer disease**	27	54	7

**Table 2:** Detailed information on the most important 11β-HSD1 inhibitors.

	**IUPAC Name**	**Molecular Formula**	**Chemical Structure Depiction**	**URL**
ABT-384	4-[[2-methyl-2-[4-[5-(trifluoromethyl)pyridin-2-yl]piperazin-1-yl]propanoyl]amino]adamantane-1-carboxamide	C_25_H_34_F_3_N_5_O_2_	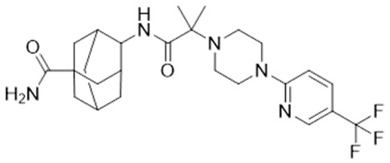	https://pubchem.ncbi.nlm.nih.gov/compound/Abt-384 (21 January 2025)
Xanamem UE2343	[(1*R*,5*S*)-3-hydroxy-3-pyrimidin-2-yl-8-azabicyclo [3.2.1]octan-8-yl]-[5-(1*H*-pyrazol-4-yl)thiophen-3-yl]methanone	C_19_H_19_N_5_O_2_S	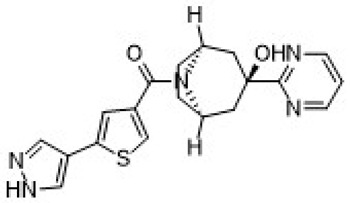	https://pubchem.ncbi.nlm.nih.gov/compound/137530063 (21 January 2025)
UE2316	(5-(1H-pyrazol-4-yl)thiophen-3-yl)(4-(2-chlorophenyl)-4-fluoropiperidin-1-yl)methanone	C_19_H_17_ClFN_3_OS	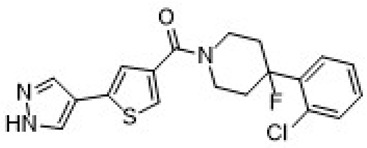	https://www.medkoo.com/products/50127 (21 January 2025)

**Table 3:** Summary of the key points from different studies across various categories.

Category	Study	Key Findings
**Basic Research on 11β-HSD1 Function**	Herbert and Lucassen [13]	11β-HSD1 inhibition protects neurons and improves cognition in aging models. 11β-HSD2 induction protects cerebellar neurons, showing dual roles in glucocorticoid metabolism.
	de Quervain Dominique [14]	Rare HSD11B1 variant with reduced 11β-HSD1 activity increases AD risk sixfold.
**Polymorphisms of HSD11B1 Gene**	de Quervain Dominique [14]	A rare rs846911 allele found in 2.9% of AD patients vs. 0.5% of controls, suggesting that genetic variants may be predisposed to AD.
	Gregory et al. [15]; Smit et al. [16]	High prevalence of 83,557 insA polymorphism in 6105 subjects, but no significant correlation with dementia incidence.
	Deary et al. [17]	No association between rs12086634-G/T, rs846910-A/G, and cognitive variation in a cohort at ages 11 and 79.
**Preclinical Evidence Supporting 11β-HSD1 Inhibition**	Li et al. [18]	Inhibition or knockdown of 11β-HSD1 in AD animal models prevents cognitive impairment and hippocampal damage.
	Chowdhury et al. [19]	11β-HSD1 overexpression in cell models results in reduced cell proliferation and increased apoptosis.
	Wang et al. [20]	Akebia saponin D (ASD) reduces corticosterone levels but does not directly inhibit 11β-HSD1.
**Clinical Trials of 11β-HSD1 Inhibitors**	Marek et al. [21]; Katz et al. [22]	No cognitive improvement in AD patients despite the effective inhibition of 11β-HSD1 by ABT-384.
	Webster et al. [23]; Taylor et al. [24]	Xanamem shows favorable brain penetration and safety. In a Phase II trial, it improves cognition in patients with tauopathy biomarkers (pTau181).
	Dodd et al. [12]	In Phase II, 10 mg/day Xanamem did not achieve statistically significant results, but new data on 20 mg/day dose may yield cognitive protection.
**Novel and Optimized 11β-HSD1 Inhibitors**	Leiva et al. [25]	Developed pyrrolidine-based inhibitors improving memory and learning in animal models.
	Puigoriol-Illamola et al. [26,27]	RL-118 alleviates cognitive impairments in aging mice, reducing neuroinflammation and glucocorticoid excess.
	Sooy et al. [28]	UE2316 reduces amyloid plaque burden and improved memory in Tg2576 mice by restoring glucocorticoid and mineralocorticoid receptor balance.
	Mohler et al. [29]	Two novel inhibitors (A-918446, A-801195) significantly improve short-term memory in rats, inhibiting cortisol production and enhancing cognition.
	Canet et al. [30]	Various 11β-HSD1 inhibitors show consistent memory improvements and reductions in amyloid plaques across animal models.
